# The impact of austerity on the health workforce and the achievement of human resources for health policies in Ireland (2008–2014)

**DOI:** 10.1186/s12960-017-0230-3

**Published:** 2017-09-11

**Authors:** Des Williams, Steve Thomas

**Affiliations:** 1 Workforce Planning, Analytics & Informatics, National HR Directorate, Health Service Executive, Swords Business Campus, Balheary Road Swords, Dublin, Ireland; 2 Centre for Health Policy and Management, School of Medicine, Trinity College Dublin, Dublin, Ireland

**Keywords:** Human resources, Austerity, Skill mix, Health policy, Cost savings, Recruitment moratorium

## Abstract

**Background:**

The global economic crisis saw recessionary conditions in most EU countries. Ireland’s severe recession produced pro-cyclical health spending cuts. Yet, human resources for health (HRH) are the most critical of inputs into a health system and an important economic driver. The aim of this article is to evaluate how the Irish health system coped with austerity in relation to HRH and whether austerity allowed and/or facilitated the implementation of HRH policy.

**Methods:**

The authors employed a quantitative longitudinal trend analysis over the period 2008 to 2014 with Health Service Executive (HSE) staff database as the principal source. For the purpose of this study, heath service employment is defined as directly employed whole-time equivalent public service staffing in the HSE and other government agencies. The authors also examined the heath sector pay bill and sought to establish linkages between the main staff database and pay expenditure, as given in the HSE Annual Accounts and Financial Statements (AFS), and key HRH policies.

**Results:**

The actual cut in total whole-time equivalent (WTE) of directly employed health services human resources over the period 2008 to 2014 was 8027 WTE, a reduction of 7.2% but substantially less than government claims. There was a degree of relative protection for frontline staffing decreasing by 2.9% between 2008 and 2014 and far less than the 18.5% reduction in other staff. Staff exempted from the general moratorium also increased by a combined 12.6%. Counter to stated policy, the decline in staffing of non-acute care was over double than in acute care. Further, the reduction in directly employed staff was to a great extent matched by a marked increase in agency spending.

**Conclusions:**

The cuts forced substantial HRH reductions and yet there was some success in pursuing policy goals, such as increasing the frontline workforce while reducing support staff and protection of some cadres. Nevertheless, other policies failed such as moving staff away from acute settings and the claimed financial savings were substantially offset by overtime payments ﻿and the need to hire more expensive agency workers. There was also substantial demotivation of staff as a consequence of the changes.

## Background

The global economic crisis (GEC) saw recessionary conditions in most EU countries. Arie [1] asserts that across the board austerity cuts have brought health systems to the brink. For Portugal, Ireland, Greece and Spain, possibly the worst affected countries in the EU, the crisis produced a change of Government, cuts to health budgets and an efficiency drive in the public sector [2]. Ireland’s GDP shrank by 7% in 2009, the greatest contraction outside of the Balkans, and extended longer than many other countries. Ireland’s severe recession produced pro-cyclical health spending cuts [3, 4], as government cut spending in an attempt to close the huge deficit caused by the collapse in its revenue as a result of the economic crash. Only Greece’s health service suffered more in terms of austerity cuts to the health service [5].

Without ring-fencing of the health budget cuts impact on human resources for health through both restricting supply, impacting on motivation and affecting the work environment [1, 6]. Yet, human resource for health (HRH) are categorised by the World Health Organization (WHO) as the most critical of inputs into a health system whose performance is dependent on the capacity and morale of staff who provide and manage care [7]. Official statistics put the fall in public sector and health service employment in Ireland at 9.6 and 11.9% respectively [8]. The cuts in health care staffing coincided with economic austerity and given that such staffing changes were so marked and counter to the prevailing trends of large increases in staffing (*prior to 2008*), the authors believe that the sole cause of the decrease stands out as being economic austerity. Prior to this, the Irish health workforce had grown by 13,000 staff between December 2004 and December 2007.

More broadly, HRH are an important economic driver with just under 6% or over 1 in 20 of the overall workforce engaged in the health sector [9]. Reeves et al. demonstrate by analysing government expenditure, before and after the GEC, across the EU that public expenditure generates an economic multiplier of 1.61 and that in the case of health that was a vigorous 4.3 [10]. The explanation being that this local expenditure stimulated the domestic economy because the bulk of the expenditure is on pay. This means cuts dampened the local economy and exacerbated the crisis.

Some authors have also suggested that a period of crisis can create the environment to enforce reforms that have lain in wait [11, 12]. The aim of this article is to evaluate how the Irish health system coped with austerity in relation to HRH and whether austerity allowed and/or facilitated the implementation of HRH policy. Secondarily, the authors reflect on the public sector recruitment ban as an effective instrument for controlling costs while meeting priorities. Consequently, the paper contributes new insights on how austerity led changes in HRH have impacted the Irish health service thus providing learning for countries facing similar situations.

### Context

The period 2007 to 2010 saw not only international austerity but also, in Ireland, a spectacular economic crash with a bank bailout, leading to the intervention of the EU/IMF troika with Ireland passing though the gravest economic crisis in its history [13], where austerity has led to pro-cyclical cuts to health services [3]. This has resulted in fiscal instability requiring “strategic consolidation and efficiency gains to fit the declining health sector budget” [14]. As defined by Blythe “Austerity is a form of *voluntary* deflation in which the economy adjusts through the reduction of wages, prices, and public spending to restore competitiveness which is (supposedly) best achieved by cutting the state’s budget, debts and deficits” [15]. Ideally, economies engage in counter-cyclical policies to stimulate the economy [16]. However, against the climate of economic crisis, Ireland announced spending reduction measures totalling €16.4 billion, in the years between 2008 and 2013, which was sufficient to deliver a €6.3 billion or 13% reduction in net government expenditure. Within the total public sector, almost half (43%) of the reported staffing cuts was delivered by the health service [8]. With the greatest numbers employed, pay measures were relatively more important in Health and Education, with the two Departments contributing €2.5 billion in pay consolidation. These measures have been central to safeguarding the stability of public finances and all sectors of the public economy contracted in monetary and employment terms since the bank guarantee. Having peaked at 9.9% of GDP in 2009 and sat just below the 8.9% OECD average at 8.1% of GDP for 2013 [5], health spending has fallen back in recent years. From 2008 to 2013 GDP fell by 17.9% and Government expenditure on healthcare fell by 17.3% or €2.8 billion to €12.8 billion over the same period.

#### Irish health policy

The government policy document “Future health, A Strategic Framework for Reform of the Health Service 2012-2015” [17] was the vehicle to implement several commitments. Future Health recommended the implementation of large-scale transformational change within the Health Service Executive (HSE) in an attempt to establish a single-tier health system in Ireland. Furthermore the transformational change of the health service identified several pillars of reform aimed at restructuring across primary community and hospital sectors to establish the basis for development of a more equitable single-tier community-based healthcare system [17].

In relation to HRH, key policy goals of government were [17]:

1. To “move away from the current hospital-centric model of care towards a new model of integrated care which treats patients at the lowest level of complexity that is safe, timely, efficient, and as close to home as possible” (pg iii)
2. To shift personnel to direct care personnel and away from support functions. “The clear focus of the reforms will be on the development and improvement of frontline services” (pg 19)
3. To produce financial reform as a key driver in promoting efficiencies in the system through a system wide pay and financial management system yielding cost savings and efficiencies

The key control instrument to support the reduction of public sector numbers in the health sector was the general moratorium on recruitment and promotions operated from March 2009 [18] which states “this moratorium is a key central feature….on saving measures on public service employment”. It goes on to exempt consultants, therapists, ambulance staff and social workers from the moratorium, because there was already recognised to be an unhelpful scarcity in these cadres, and specifies growth targets for these grades. It also stipulated that 2000 whole-time equivalents (WTE) will be transferred from acute to non-acute services to facilitate integrated care and set an initial ceiling for employment at 111,800 WTE for 2009. The moratorium was still in place at 31 December 2014, the end evaluation point for this article.

The moratorium on recruitment was followed by a targeted Voluntary Early Retirement Scheme [19] which saw in excess of 1600 WTE [20] in management, administrative and support staff exit from non-frontline or support roles.

## Methods

The authors adopted a quantitative approach since available data on workforce, skill mix, pay statistics, and the allocation of resources are quantitative. Furthermore, with the exception of headline figures, there has been no in-depth quantitative analysis of the health workforce configuration. This research employed a quantitative longitudinal trend analysis over the period 2008 to 2014 with HSE staff database interrogation as the principal method. Pre-collected data has been recognised as a valid instrument for this empiricist approach to research [21]. Since 1990, health service employment has been reported through a database^1^ known as the Health Services Personnel Census (HSPC), at first collected by the Department of Health (DoH) and then, upon establishment, the HSE. Access to this complete dataset was granted by the HSE to the researcher and the first phase of the study involved data extraction from the existing heath service personnel census database.

For the purpose of this study, health service employment is defined as directly employed whole-time equivalent public service staffing in the HSE and other agencies encompassed by section 38^2^ of the Heath Act (2004), as covered by the public service employment numbers (Department of Public Expenditure and Reform, 2014) and the Government Employment Control Frameworks (HSE, 2009).

The authors also examined the heath sector pay bill and sought to establish linkages between the main WTE dataset and pay expenditure. Pay expenditure is published in the HSE Annual Accounts and Financial Statements (AFS) and categorised as follows: Direct pay costs (basic, overtime, on call, allowances, weekend/public holiday, night shift, employers PRSI, arrears/other), agency and “superannuation”. Direct pay is the cost of paying direct employees. The combination of direct pay and agency gives the “service delivery pay costs”. The final element of pay expenditure is superannuation and includes normal pension payments to retired employees and lump sums paid on death, retirement or redundancy. Monthly pay data were collated on an annual basis in Excel on the basis of the pay elements previously referred to.

These data allowed the expenditure on the various direct pay components (basic, allowances, on-call, overtime, night shift, weekend/public holiday, arrears/other and night shift) to be tracked over time and to quantify key change. This pay expenditure also included two further components which are not related to direct employment, superannuation (pension) payments and expenditure on agency. All costs are given in constant Euro.

Finally, in order to fully evaluate the skill mix, an additional pre-crisis comparative date was sought when the total available HRH was homogeneous to December 2014. As the totals are almost identical, it is possible to profile the staffing changes over the two dates on a like-for-like basis. This date was March 2006 (103,262 WTE) where there was less than one half percentage less whole-time equivalent (WTE) HRH.

By combining clustered organisation units and grouped grades, the researcher developed an analysis strategy for segmenting, viewing and understanding data in a database, cutting the data into smaller parts, and repeating this process in various views in order to arrive at the right level of detail for analysis and to present data in new and diverse perspectives [22].

This includes presenting data across the service divisions, focusing on service delivery areas of Acute Services and﻿ Community﻿ Services (Mental Health, Primary Care and Social Care together with Child & Family Services) which cover 95% of directly employed staff.

This process has also been followed at a staff group level to allow for clearer visibility over the six staff categories but within a manageable framework to cluster the 600 health service grades into 16 distinct groups, so as to allow for analysis by profession and between grades which are of direct service (frontline) and support grades (support). This allows for distinction between consultants and non-consultant doctors, therapists, nurses and nurse managers, support and care grades, administrative and management and other professionals matching categories used for workforce planning [23].

Business Objects (BO) software was used to analyse and evaluate the large amounts of WTE-related data. BO is a front-end business intelligence software which establishes a database link and enables query construction, analysis and presentation. Ethical approval was sought from, and granted by, the School of Medicine Ethics Committee in Trinity College, Dublin.

### Corrections and limitations

On 1 January 2014, the Child & Family Division of the Department of Health and Children transferred its 3500 staff to the Child and Family Agency (CFA) 2014. According Bell et al. “missing data can reduce the power and efficiency of a study but, unfortunately, can also lead to biased results” [24]. The last observation carried forwards (LOCF) method is a common approach to resolving the missing data issue [25]. In order to remove a completely as possible any bias in the data and present information on a consistent basis, the authors rolled-forwards the 3465 WTE recorded at December 2013 for each quarter in 2014.^3^ A further adjustment has been made to include new graduate nurses, support and care staff on reduced rates of pay, accounting for an additional 1536 WTE at 31 December 2014 which were not reported in the official dataset but are part of the workforce.

Certain inconsistencies were identified between human resources and pay data source systems.

- Annual financial data is built on the principle of cumulative monthly data as opposed to employed at month end. To allow for this, average WTE for each year was used rather than year-end figure in order relate pay to WTE and to track average pay movements
- This average WTE was adjusted to exclude 13 section 38 voluntary public ﻿health agencies not covered by the pay data for historical accounting reasons.
- Hours worked through agency and overtime are not expressed in HRH WTE but in cost terms only. This information is not available through the national HR systems.

## Results

The actual cut in total WTE of directly employed health service human resources over the period 2008 to 2014 is 8027 WTE (a reduction of 7.2% and not almost 12% according to  published official sources). The difference between the official figures and the true figure is 3465 transferred staff in the Child & Family Agency together with 1536 WTE graduate nurses, care and support “intern” staff which went unreported in official returns up to 31 December 2014, as shown in the aggregates of Table 1. Possible reasons for this over-reporting of lost staff will be reviewed later.

**Table 1:** Overall and categorised staffing changes 2008–2014

Staff group	Total (2008)	2008%	Total (2014)	2014%	Change	% Change
Therapists (Physio, OT, SLT)	3209	2.9%	3769	3.7%	*+ 560*	+ 17.4%
Nurse specialist	869	0.8%	1332	1.3%	*+ 463*	+ 53.3%
NCHDs	4924	4.4%	5302	5.2%	*+ 378*	+ 7.7%
Consultants	2292	2.1%	2635	2.6%	*+ 343*	+ 15.0%
Ambulance	1303	1.2%	1556	1.5%	*+ 253*	+ 19.4%
HSCPs other	12,485	11.3%	12,526	12.2%	*+ 42*	+ 0.3%
Public health nurses	1465	1.3%	1466	1.4%	*+ 1*	+ 0.1%
Other medical and dental	887	0.8%	881	0.9%	*− 6*	− 0.7%
Management	1257	1.1%	1225	1.2%	*− 32*	− 2.5%
Other nursing	361	0.3%	289	0.3%	*− 72*	− 20.0%
Nurse management	7393	6.7%	6627	6.5%	*− 766*	− 10.4%
Care staff	17,404	15.7%	16,538	16.1%	*− 865*	− 5.0%
Clerical and secretarial	16,671	15.0%	14,351	14.0%	*− 2 320*	− 13.9%
Staff nursing	27,810	25.1%	24,829	24.2%	*− 2 981*	− 10.7%
Support services	12,489	11.3%	9466	9.2%	*− 3 024*	− 24.2%
Total	110,819	100%	102,792	100%	*− 8 027*	− 7.2%

### Health policy goals

**Fig. 1:**
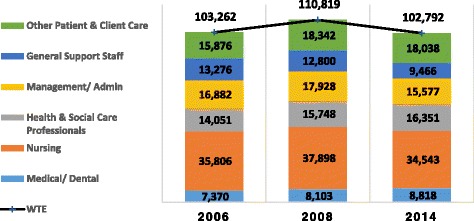
Homogeneous skill mix profile by staff category 2006 and 2014

The protection of front-line services was a stated health policy for the government. The evidence suggests that there was a degree of relative protection for frontline staffing. It decreased between 2008 and 2014 by 2.9% (or 2341 staff) but this was far less than the 18.5% reduction in other staff, with just under 5700 WTE losses. The latter is made up of some reduction in management and administration but a sharp reduction in support staff (see Fig. 1). The profile change also shows staff nursing fall substantially but increase for nurse specialists and therapists (Table 1). As stated earlier, certain categories of staff were exempted from the moratorium. The introduction of this strategic HRH policy contributed to a combined + 12.6% increase in these grades. Posts not exempted fell by 10% or greater than the average 8%.

One of the key policy goals highlighted was to shift the balance and numbers of staff towards ﻿community﻿ based care to improve both cost-saving, integration and efficiency. At the outset in 2008, community services had 1945 WTE more staff than in acute care but by December 2014; the balance was reversed and there were 2153 more acute services staff than in the non-acute sector. Admittedly, staff were lost from both areas but it is interesting that the decline in staff in non-acute care was over double that in acute care. Having said that, the reductions were in both cases substantial and it may be that more significant staffing losses in hospitals would have required substantial reorganisation rather than just a reallocation of workload across the remaining staff. The trends may also reflect the greater power and political importance of hospitals. There was media attention on the growing numbers of patients on trolleys in hospitals and on increased waiting lists for hospital treatments. This may itself have meant there was less action taken in relation to hospitals which overspent.

The third policy objective was related to cost control and efficiency. The cuts to pay and staff numbers brought the pay bill down by 11%. However, this does not take into account the cost of temporay agency staff and the increased cost of superannuation﻿ (﻿*staff pensions*)﻿. When these are included, only a 3.4% savings is realised.

**Fig. 2:**
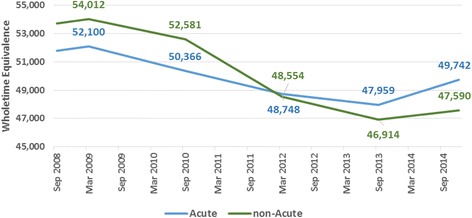
Acute and non-acute staffing 2008–2014

**Fig. 3:**
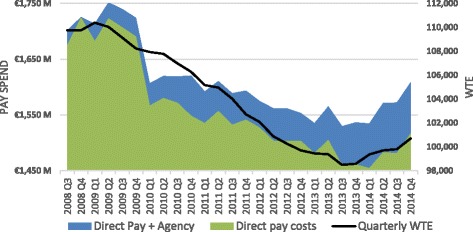
Stacked direct pay and agency, WTE 2008–2014

As directly employed staff numbers fell, there was also a marked increase in agency staff spend from Q1 2010 onwards in particular, as seen in Fig. 2. This highlights some of the false economies of reducing direct staffing (Fig. 3).

The combination of direct pay decreases and agency pay increases meant that overall savings to service delivery pays costs were much reduced. The €267.7-million increase in agency spend, which had ballooned by 320%, diminished the direct pay spend savings to €476.8 million. Furthermore, because of the voluntary retrenchment packages which many staff approaching retirement took, the pension spend increased by €226.8 million (or over 44%). This was a very high cost to pay and the resources spent on agency staff and increased pensions would have allowed the payment of an e﻿s﻿timated additional 3800 staff. Adopting these strategies meant that payroll costs were in effect only 3.4% lower, despite a 6.5% cut to pay rates and a 7.2% fall in HRH numbers.

Furthermore, in terms of overall system performance, there is some evidence to suggest that the Irish system initially became more efficient in the austerity era in terms of higher throughput in hospitals because of more day-cases while maintaining the same length of stay in hospitals with fewer resources^6^. Nevertheless, from 2013 onwards, the cumulative impact of fewer finances, reduced human resources and continued closure of acute beds led to the system reducing its productivity incurring rapidly rising waiting lists and cutting of home-help services^4^.

## Discussion

The analysis done shows that headline figures for staffing changes in the Irish health care system fall short of the reality. This may have come from a desire to overstate the size of adjustment to lessen future funding cuts. However, it is also clear that resources were not well deployed and cost savings were in fact false economies and this may particularly relate to imposing a moratorium and the consequent need to hire temporary staff.

Australian research titled “Death by a thousand cuts” [26] evaluates the impact of applying across-the-board public sector cuts came to the conclusion that not only did cuts reduce the quality and availability of services, they also caused long-term damage to the institutions of government. In the context of cutting workforce numbers, they found that decreasing retention rates led to substantial losses of institutional knowledge and skills, exacerbating problems with staffing and skill shortages. “Evidence suggests that recruitment freezes are probably the most detrimental approach to downsizing, because they are indiscriminate and limit the ability of organisations to restructure and re-skill” (p6) [27].

Certainly, the deep cuts in staffing profoundly affected morale as revealed by the first ever Irish Health Services Staff Survey was undertaken at the end of 2014 and had a low 7.1% response rate. The survey had some positive findings, particularly in relation to their own roles. Nonetheless, staff were generally pessimistic about the future and dissatisfied with HSE as an employer and they did not feel that the health system puts patient care as its top priority. In relation to leadership, they did not feel that their manager delegated well, gave feedback or listened to ideas and suggestions [28]. Indeed, many staff did not feel their work was valued (p31), were not satisfied with their salary or their support from line managers (p11).

Ireland was typical in terms of countries hard hit by austerity in imposing public sector wage cuts in response to the crisis [29]. Nevertheless, it was more unusual in losing significant numbers of staff through voluntary retrenchment and this may well have been an inevitability of the combined depth and length of the recession^3^. In keeping with several other European countries in crisis, it was committed to prioritising primary care staffing [29], but in common with Greece, it was not able to achieve this despite its ongoing commitment to universalism. A key emphasis in many countries is also to move away from a reliance on physicians in primary care [30, 31]. However, it appears that this was not a focus of policy reform for Ireland.

## Conclusions

Budget cuts forced substantial HRH changes. Yet within that there was some success in pursuing policy goals, such as increasing the frontline workforce while reducing support staff and protection of some identified and prioritised cadres. But there was resistance to moving staffing away from acute settings which ran counter to official government policy. Also there was a deep sense of demotivation of staff as a consequence of all the changes. Both of these factors may impact on government’s ability to move towards universal health care as outlined in Future Health.

Further, some of the financial savings produced were real but others were not because of the moratorium and the need to hire more expensive temporary staff. Indeed, more efficient use could have been made of the resources deployed on agency staff and through the offered retirement packages. Such strategies may have placed more strain on the system than was necessary.

## Abbreviations

AFS: Accounts and Financial Statements
BO: Business Objects
CFA: Child and Family Agency
DOH: Department of Health
EU: European Union
GDP: Gross domestic product
GEC: Global economic crisis
HR: Human resources
HRH: Human resources for health
HSE: Health Service Executive
HSPC: Health Services Personnel Census
IMF: International Monetary Fund
LOCF: Last Observation Carried Forward
OECD: Organisation for Economic Co-operation and Development
WTE: Whole-time equivalents

## Footnotes

1 The database has 1.3 m records—covering staffing by grade, contract type, gender and location.

2 Section 38 of the health act relates to the direct provisions of a service. All 18 voluntary hospitals and 35 mainly disability agencies fall into the category.

3 These figures closely relate to the increase in DCYA figures for 2014 (<80 WTE) as published by Department of Public Expenditure and Reform (Department of Public Expenditure and Reform, 2014) allowing the authors to have confidence in the figures.
